# Assessing the Quality of Evidence Regarding Permanently Listed Digital Health Applications (DiGAs) in Germany: A Scoping Review

**DOI:** 10.3390/healthcare14152279

**Published:** 2026-07-26

**Authors:** Sebastian Zapp, Annabelle Mielitz, Ute von Jan, Urs-Vito Albrecht

**Affiliations:** 1 Department of Digital Medicine, Medical School OWL, Bielefeld University, Universitätsstraße 25, 33615 Bielefeld, Germany; sebastian.zapp@uni-bielefeld.de (S.Z.); annabelle.mielitz@uni-bielefeld.de (A.M.); 2 Peter L. Reichertz Institute for Medical Informatics of TU Braunschweig and Hannover Medical School, Hannover Medical School, Carl-Neuberg-Str. 1, 30625 Hannover, Germany; ute.von.jan@plri.de

**Keywords:** digital health applications (DiGAs), evidence quality, scoping review

## Abstract

**Highlights:**

**What are the main findings?**

- The literature consistently identifies methodological weaknesses in studies supporting permanently listed DiGAs, particularly concerning blinding, dropout rates, and study design.
- Many DiGA evaluations are based on small study populations, patient-reported outcomes, and passive control groups, potentially limiting the robustness of the evidence.

**What are the implications of the main findings?**

- Improving methodological quality and reporting standards is essential to strengthen confidence in the evidence supporting DiGAs.
- Sustainable integration of DiGAs into routine care requires not only evidence pathways that combine methodological rigor with ongoing generation of real-world data but also viable approaches to addressing the financial and structural challenges that DiGA manufacturers encounter during the study-based evidence generation phase.

**Abstract:**

**Background/Objectives**: Germany’s 2019 Digital Healthcare Act allows manufacturers to integrate digital health applications (DiGAs) into statutory health insurance if they demonstrate a medical benefit or patient-relevant structural or procedural improvement, substantiated by comparative studies. However, existing reviews indicate that the quality of evidence in these studies is inconsistent. **Methods**: A scoping review following the PRISMA-ScR guidelines was conducted using MEDLINE on 16 December 2024, via PubMed, Scopus, EMBASE, and Cochrane Library. Studies published between 2020 and 2024 assessing DiGA evaluation tools, study quality, and the strength of evidence were included, while non-DiGA studies, telemedicine research, and register-based studies were excluded. **Results**: Out of the 4628 publications screened, 16 met the inclusion criteria. Across the nine dimensions of evidence quality, deficits were most frequently identified in relation to validity (nine publications), transparency (seven), objectivity (six), consistency (five), and reliability (five), followed by relevance, generalizability, and accuracy (four each) and timeliness (two). **Conclusions**: The findings highlight substantial limitations in the quality of DiGA studies, including high dropout rates, a lack of blinding, gender imbalance, a high risk of bias, small sample sizes, and biased control-group designs. Additionally, DiGA usage durations were often shorter than recommended. Strengthening the evidence base for DiGAs requires balancing methodological rigor with the practical realities of digital health development. Sustainable integration into routine care depends on feasible evaluation pathways that enable continuous, real-world evidence generation and address the financial and operational challenges of high-quality evaluations.

## 1. Introduction

### 1.1. Background

In Germany, “Digitale Gesundheitsanwendungen” (DiGAs) are CE-marked medical devices used either independently by patients or jointly with healthcare professionals [1]. These applications are built on digital technologies [1] and include a variety of software formats, such as mobile apps, web-based platforms, and other digital tools designed for medical purposes. DiGAs are intended to detect, monitor, treat, or alleviate diseases and support the management of injuries or disabilities [1]. Germany has a statutory health insurance system that covers the majority of the population. Within this system, DiGAs can be prescribed by licensed physicians or psychotherapists and, if listed in the official DiGA directory, are reimbursed by the insurance funds. Since the Digital Healthcare Act (Digitale-Versorgung-Gesetz, DVG) was enacted in 2019 [2], DiGAs therefore represent a new reimbursable category of “prescription apps” within routine care. The regulatory framework, overseen by the Federal Institute for Drugs and Medical Devices (BfArM), includes a structured approval process to enable rapid integration of innovative digital therapies into routine care. This “fast-track” process allows for provisional listing in the DiGA directory (DiGA-Verzeichnis, DV) for up to 12 months—extendable to 24 months—during which manufacturers must submit scientific evidence demonstrating a positive impact on care [3]. Eligibility for the directory requires that DiGAs be CE-marked as Class I or IIa medical devices under the EU Medical Device Regulation (MDR) and demonstrate measurable improvements in patient care. Following the enactment of the Digital Act (Digital-Gesetz, DigiG) on March 26, 2024, the scope of digital health applications (DiGAs) was broadened to include MDR (Medical Device Regulation) risk class IIb medical devices, provided they comply with §33a SGB V, the Digital Health Application Ordinance (Digitale-Gesundheitsanwendungen-Verordnung, DiGAV)**,** and the DiGA Guidelines [3]. However, it is important to note that the fast-track process is distinct from the general EU-wide CE-marking procedure and is designed to enable rapid integration of innovative digital therapies into everyday care.

Accepted evidence typically involves quantitative, comparative study designs—such as randomized controlled trials (RCTs), cohort studies, or meta-analyses—demonstrating direct medical benefits or patient-relevant structural or procedural improvements [3]. Non-quantitative studies, case reports, and expert opinions are excluded. In addition to clinical efficacy, applications must meet stringent safety, technical performance, usability, data protection, and interoperability standards.

The BfArM’s evaluation is document-based and coordinated with the Federal Data Protection Officer (BfDI) to ensure compliance with the General Data Protection Regulation and national standards such as the Digital Health Application Ordinance (§§ 4–6) and the Digital Care and Nursing Modernisation Act [4]. These requirements are intended to uphold high standards of reliability, functionality, and security while fostering innovation. Germany’s DiGA framework has been internationally recognized for its structured integration of evidence-based digital therapeutics into public healthcare. Unlike traditional interventions, DiGAs provide benefits such as shorter innovation cycles, personalization, and scalability, making them especially valuable in managing chronic diseases and improving access to care [5].

### 1.2. Definition of “Quality of Evidence”

High-quality evidence is critical for effective health, policy, and scientific research [6]; decision-making in general; and assurance of the safety, effectiveness, and suitability of digital healthcare interventions. It determines how confidently findings can guide actions and clinical decisions, shape interventions, or support policy changes [7]. Evaluating evidence quality involves assessing various methodological and contextual dimensions that influence the credibility and applicability of results [8]. To define evidence quality, this scoping review builds on the appraisal framework developed by Spencer et al. [9] for the UK Cabinet Office Strategy Unit and the National Centre for Social Research to establish explicit standards for evaluating research quality. The framework specifies nine dimensions of quality: relevance, reliability, validity, objectivity, accuracy, transparency, timeliness, generalizability, and consistency [9]. According to Spencer et al. [9], relevance (REL) refers to how evidence directly addresses the corresponding research questions and policy concerns. Reliability (RIL) involves the robustness and replicability of data collection and analysis procedures [10], while validity (VAL) concerns the credibility and plausibility of the findings in terms of reflecting participants’ perspectives. Objectivity (OBJ) emphasizes minimizing researcher bias and is supported by clear documentation of interpretive processes. Accuracy (ACC) pertains to the precision and correctness of data representation. Transparency (TRA) denotes the clarity with which methodological decisions and analytic pathways are reported. Timeliness (TIM) reflects whether the evidence used to inform decisions is current and appropriate within its respective context. Generalizability (GEN), though nuanced in qualitative work, refers to the extent to which findings may be transferable to other settings. Finally, consistency (CON) relates to the coherence of findings across sources and the stability of interpretations throughout the analysis. These dimensions collectively guide evaluative rigor and help assess whether evidence is fit for informing decisions, particularly in policy-making, healthcare, and research contexts. By examining each of these dimensions, users can critically appraise the strengths and limitations of evidence, ensuring that conclusions drawn are based on sound, trustworthy information [9].

### 1.3. Aim of This Paper

Given the pivotal role of evidence in the regulatory model for DiGAs, it is essential to critically examine the quality of the scientific evidence supporting the permanent approval of DiGAs. This scoping review is part of a broader project examining evidence generation for digital health applications (DiGAs). While this review summarizes the literature on evidence quality and recurring methodological challenges, there is also a complementary study directly assessing the methodological quality and risk of bias of the clinical studies supporting permanent DiGA listing. In contrast, our scoping review focuses on mapping and synthesizing the existing literature on evidence quality and identifying recurring methodological limitations reported across publications. The two manuscripts differ in their objectives, methodologies, and analytical scope and are reported independently to ensure clarity, methodological transparency, and appropriate interpretation of the findings.

This background leads to the central research question: how is the quality of scientific evidence for permanently listed digital health applications (DiGAs) assessed in the literature, and which methodological shortcomings are repeatedly identified?

## 2. Materials and Methods

### 2.1. Overview

A preliminary search was conducted on MEDLINE via PubMed and Google Scholar to identify relevant keywords from titles and abstracts. Based on this exploratory analysis, a comprehensive search for publications published between 2020 and 2024 was performed across MEDLINE via PubMed, Embase, Scopus, and the Cochrane Library using the following search string: (quality of evidence OR evidence) AND (mobile health applications OR DiGA OR DiHA). This search strategy was designed to identify publications explicitly addressing evidence quality in the context of DiGAs rather than studies evaluating the effectiveness of individual digital health applications. The retrieved records were downloaded, and duplicates were removed using Rayyan (Rayyan Systems Inc., Cambridge, MA, USA). Systematic screening was then performed by reviewing titles and abstracts. Studies that did not meet the inclusion criteria described below were removed. If a study’s title and abstract did not indicate whether it should be included, the decision was based on a review of the full text. The preliminary and systematic searches, screening, study selection, full-text assessment, and data extraction were conducted by a single reviewer. To assess the consistency of screening decisions, a retrospective intra-rater reliability assessment was conducted after completion of the review process. The results of this assessment are reported in Section 4.5. Although dual independent screening is recommended for scoping reviews, resource constraints prevented its implementation. The publications included were examined for aspects related to evidence quality and thematically assigned to the following nine dimensions of evidence quality [9]: relevance, reliability, validity, objectivity, accuracy, transparency, timeliness, generalizability, and consistency [9]. The methodological aspects were assigned to the nine evidence-quality dimensions by the authors based on the conceptual definitions proposed by Spencer et al. [9], and this scheme was applied consistently across all included publications. Each publication could be assigned to multiple evidence-quality dimensions. The assignment was performed deductively using the framework proposed by Spencer et al. and was based on explicit methodological strengths or weaknesses reported in the publications. A coding table was developed to ensure consistent categorization across studies. In some cases, descriptive measures were calculated (absolute and relative frequencies). The review process was reported in accordance with the PRISMA-ScR guidelines [11]. It was structured using the Arksey & O’Malley framework for structured scoping reviews and the Joanna Briggs Institute methodology for scoping reviews [12]. No review protocol was registered. While protocol registration is recommended for scoping reviews, it is not mandatory according to PRISMA-ScR guidance.

### 2.2. Concept

This scoping review is designed to systematically present potential gaps in the quality of evidence for studies of permanently listed digital health applications (DiGAs) within the German healthcare system.

### 2.3. Context

We sought publications that, while focusing on the core concept mentioned above, address the German healthcare system. The intended use of the permanently listed DiGAs is in clinical and/or outpatient settings.

### 2.4. Inclusion and Exclusion Criteria

The inclusion (Appendix A) and exclusion criteria were established through an exploratory search conducted by a single reviewer, a review of relevant publications, and internal discussions with two colleagues from the Digital Medicine Working Group at the Medical School OWL, Bielefeld University. As DiGAs have only existed since 2020, search results were limited to 2020–2024. Publications were included if they provided an overview of the strength of the evidence for the positive impact on care in studies on permanently listed DiGAs. Due to the scoping nature of this review, we considered regulatory analyses, market analyses, and opinion papers eligible when addressing methodological challenges, evidence requirements, transparency, or evaluation approaches.

We excluded studies on DiGAs that were not permanently listed in the DV, studies in languages other than German or English, studies on an irrelevant topic (e.g., health apps or the evidence quality regarding health apps outside the DV; telemedicine; and cross-sectional studies on health apps), and scoping review protocols. Additionally, case–control, cohort, and registry-based studies focusing on specific interventions and outcomes rather than a broader assessment of evidence quality were deemed unsuitable for answering the research question and thus excluded. Finally, studies that could not be retrieved in full-text format were also excluded.

## 3. Results

### 3.1. Overview

**Figure 1:**
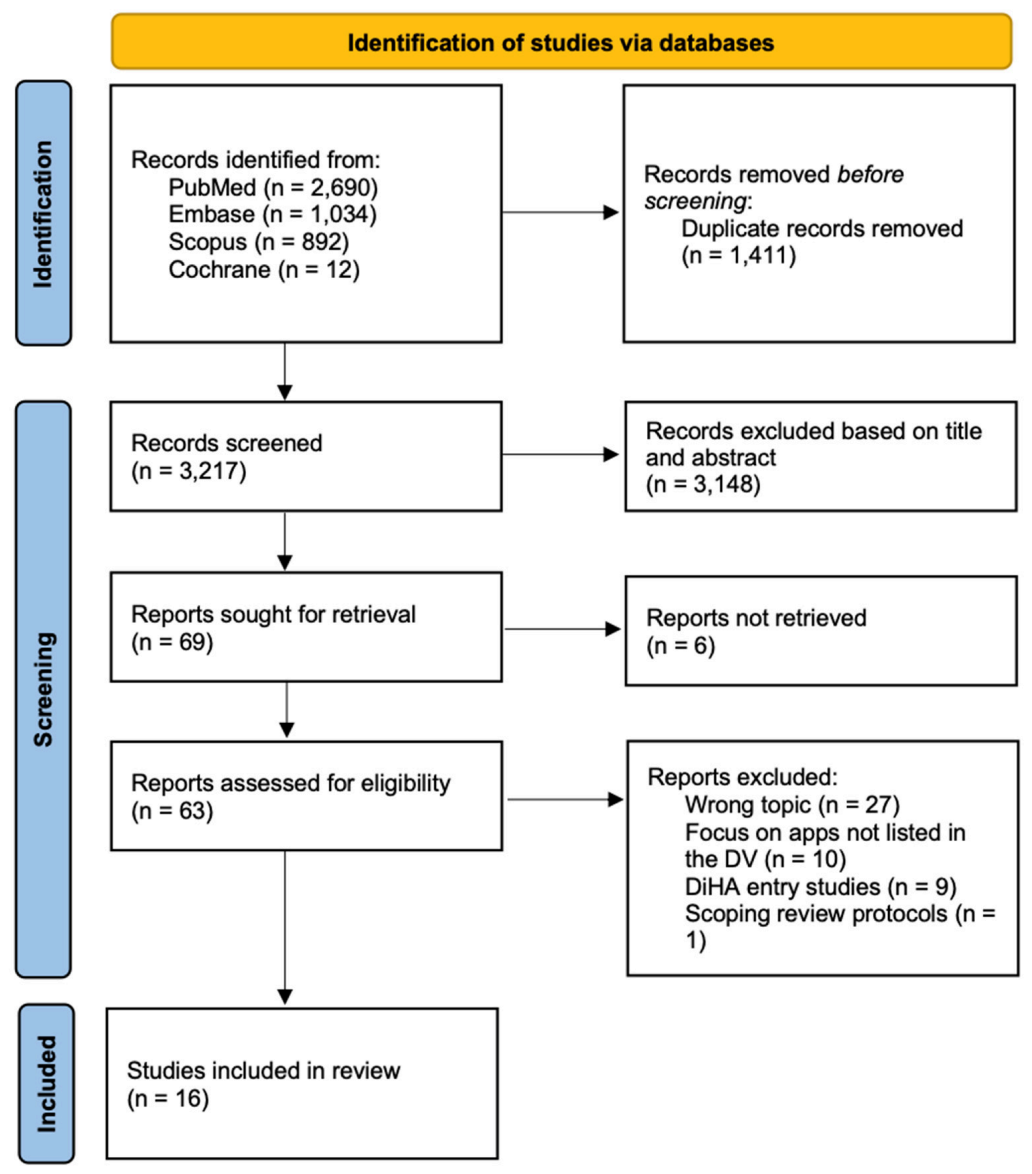
PRISMA-ScR flowchart.

The searches for the relevant literature and data extraction were conducted independently by an author on 16 December 2024. A total of 4628 entries were identified. After 1411 duplicates were removed, 3217 articles were screened. Following application of the inclusion and exclusion criteria and removal of retracted articles, 3201 articles were excluded from consideration. The remaining 16 articles were deemed suitable and thus included in the analysis (see Figure 1). Publication types were categorized based on the methodological approach reported by the authors of the studies examined. Publications with structured data extraction and predefined assessment criteria, but without fulfilling formal requirements of a systematic or scoping review, were classified as structured assessments.

Six of the publications included in the scoping review were classified as structured assessments. The other publications were classified as narrative reviews (n = 4), opinion or position papers (n = 2), or cross-sectional analyses (n = 2), with one scoping review and one mixed-methods study that combined questionnaire development with a subsequent cross-sectional analysis. Almost all these publications (n = 15) were conducted without predefined search strategies, explicit inclusion and exclusion criteria, or a PRISMA-compliant methodology. A scoping review conducted by Schreiter et al. [13] was included, but it does not report on PRISMA-ScR. A tabular overview (Table 1) was created to present the included literature in a structured manner, summarizing key characteristics such as publication year, publication type, thematic focus, and, if reported, the number of DiGA-relevant primary studies.

The following section analyzes the aspects related to evidence quality identified in these articles. We identified 12 aspects: blinding, dropout rates, control group, primary endpoint, indication, sample composition, study participants, sample size, mandatory publication, application and follow-up rate, usage duration, and evidence of medical benefits/patient-relevant structural or procedural improvements. These aspects were assigned to the nine dimensions of evidence quality, as presented in Table 2.

For each study included, Table 3 indicates whether it addresses the nine previously mentioned evidence quality dimensions; e.g., if a publication mentions a lack of blinding in DiGA-related studies, it would be considered to address the aspect of objectivity.

### 3.2. Detailed Analysis

#### 3.2.1. Objectivity (Aspect: Blinding)

In their scoping review, Schreiter et al. [13] examined three meta-analyses, 39 RCTs, and two single-arm interventions. The 39 RCTs were explicitly conducted to support the approval of DiGAs in the DV. A total of 29 (74%) of these 39 RCTs were either not blinded or lacked information on blinding. Albrecht et al. [14] also noted a lack of blinding in 14 (82%) of the 17 studies they examined. König et al. [15] stated that the studies were essentially open-label, with the control group not blinded; i.e., a placebo in the form of another DiGA was not provided. Using the RoB2 tool, in March 2022, Kolominsky-Rabas et al. [16] found that “none of the studies on the [six examined] DiGA [are] blinded” [16]. Gregor-Haack et al. describe a high risk of bias due to the “unblinded assessment of endpoints with self-administered questionnaires” [17]. They mention the possibility that a supposedly significant result could be falsely reported, even though there is no real therapeutic effect owing to inadequate blinding. In this regard, placebo DiGAs could be used as a control in studies. This pathway would allow for “blinding to be maintained” [19].

#### 3.2.2. Reliability (Aspect: Dropout Rates)

Five publications addressed the problem of high and heterogeneous dropout rates in DiGA studies [13,14,16,17,19]. Schreiter et al. reported significant “dropout rates of up to 60% in some studies” [13]. Mäder et al. [19] emphasized that dropout rates were highly variable across studies, while Gregor Haack et al. [17] noted “considerable uncertainty” in the efficacy assessment of the results due to high average dropout rates across studies. On average, study participants more often drop out of intervention groups than control groups [14,19]. There was agreement in the results reported by Kolominsky-Rabas et al. [16] and Gregor-Haack et al. [17] that the dropout rates in two studies were high and that a risk of bias could not be ruled out.

#### 3.2.3. Validity (Aspects: Control Group and Primary Endpoint)

##### Control Group

Eight publications (33%) addressed the adverse effects of passive control groups, highlighting a gap in the methodological quality of the evidence. Twenty-two (61%) of 36 studies used waiting-list control groups, and 12 (33%) compared standard therapy with an intervention group [18]. Active control groups were used in 6 of the 39 studies examined [13]. Through a closer examination of the effectiveness of two DiGA studies, König et al. [15] specifically demonstrated that a pure placebo effect due to non-treatment in the control group could not be ruled out. Lantzsch et al. [20] also addressed the issue of control groups. They referred to criticism of company health insurance funds leveled by their umbrella association, which argued that there is often no active control group but rather a passive waiting-list control group. Therefore, in the view of Lantzsch et al. [20], it was impossible to determine whether DiGAs provided a treatment advantage over conventional treatments. Albrecht et al. [14] noted that, in many studies, it is unclear whether participants in the control group had previously undergone treatment or were unfamiliar with it. This issue further enhances the risk of bias. Kolominsky-Rabas et al.’s [16] evaluation of nine DiGA studies in March 2022 showed potential bias in the waitlist control groups. Control groups having to wait for treatment without therapeutic addition in the form of a DiGA may lead to a more pessimistic assessment of the comparison group, and this eventuality could then be reflected in the patient-reported outcomes (PROs), which are predominantly used in DiGA studies as primary endpoints [16]. Goeldner et al. [18] reported that no patterns could be identified in the design of the control group relative to the DiGA indication.

##### Primary Endpoint

Most studies have a high risk of bias because they use PROs as primary endpoints [15,16,17,21]. The evidence supporting the selection of the aforementioned primary endpoints in the DiGA studies is questionable [23]. König et al. [15] explained that using PROs as a surrogate endpoint makes it “more difficult to distinguish the effect of the DiGA from other effects such as a placebo effect” [15]. A potential risk of bias arises from selecting a self-assessment instrument as the primary endpoint [16]. Fujita-Rohwerder et al. [23] noted doubts about studies’ suitability for demonstrating a positive impact on care due to endpoint selection, the timing of endpoint measurement, and the choice of control intervention. Heterogeneous selection of endpoints complicates the comparability and internal validity of the studies. A standardized approach would be more efficient, especially when DiGAs with the same indication are to be permanently listed in DV [19].

#### 3.2.4. Relevance (Aspect: Indication)

König et al. [15] describe the investigation of DiGAs in scientific studies as “challenging,” as aligning the medical indication and intervention goal can be difficult in DiGA studies because interventions in these studies are often complex and multifaceted. Furthermore, Kolominsky-Rabas et al. [16], in their analysis of DiGA studies using the RoB2 tool, found that at least some concerns about risk of bias due to deviations from the intended intervention exist in almost all DiGA studies. However, there is still no clear consensus on this issue, as Lanztsch et al. [20] found that virtually no clinical trials (n = 10/11) deviated from the intended interventions. Gregor-Haack et al. [17] report on a study for which the exact design of the intervention remains unclear. At the same time, Gregor-Haack et al. [17] also report positively on the interventions in DiGA studies on permanently included DiGAs while also highlighting that studies on applications such as Invirto raise concerns about transparency in the adaptation of digital interventions to disorder-specific symptoms, such as the personalization of exposure training for anxiety disorders [17]. Related studies are more detailed, allowing the corresponding interventions to be assessed more accurately.

#### 3.2.5. Generalizability (Aspects: Sample Composition and Study Participants)

##### Sample Composition

Four publications found that men were underrepresented in some studies [13,16,18,19]. For one DiGA, approval was limited to women at the time of the study because there were not enough men in the clinical trials, even though the study was initially designed for both genders [16]. Consequently, Mäder et al. [19] found that in the DiGA mentioned above, the majority of participants were female. Apart from this case, Goeldner et al. [18] report a lack of transparency in the DV regarding the gender distribution of study participants. They mention that only 22 (61%) of the 36 studies examined published the gender distribution of their participants [18].

##### Study Participants

As Kolominsky-Rabas et al. [16] have shown regarding the studies they examined, it was unclear whether the studies investigated the entire target population as defined by the respective codes of the International Statistical Classification of Diseases and Related Health Problems 10th Revision German Modification (ICD-10-GM) or whether they focused only on specific subgroups.

#### 3.2.6. Accuracy (Aspect: Sample Size)

Four publications reported notably small study samples—especially compared to typical RCT sample sizes—or substantial heterogeneity in sample sizes [14,18,21,25]. Albrecht et al. [14] determined there was a median of n = 215 in 17 examined studies. Chalmers et al. [21] found a median sample size of n = 171 in 53 studies. Additionally, Goeldner et al. [18] and others [14,21] noted that substantial variation in sample size limits comparability and weakens statistical conclusions. According to Koerber et al. [25], small sample sizes may not adequately reflect the reality of care. However, on a positive note, Goeldner et al. [18] reported a gradual upward trend in sample sizes in recent years (median sample size n = 139 in 2020, n = 264 in 2021, n = 187 in 2022, n = 253 in 2023, and n = 220 in 2024).

#### 3.2.7. Transparency (Aspect: Mandatory Publication)

Lantzsch et al. [20] reported potential for improvement regarding the transparency of DiGA studies, especially given that the requirements for sufficient evidence for DiGAs are comparatively low compared to those for other reimbursable medical devices [24]. Publication status affects the actual use of DiGAs by healthcare workers. Geier [27] and Dahlhausen et al. [28] reported widespread uncertainty among physicians and physiotherapists regarding therapeutic benefits, technical reliability, and clinical evidence regarding DiGAs. In fact, over 70% of surveyed clinicians reported a need for more accessible, detailed information in order to evaluate and recommend DiGAs to patients [28] adequately. These concerns are reinforced by the findings reported by Goeldner et al. [18], who found that only 37% of DiGA manufacturers provide a link in the DiGA directory to the full-text publication of their supporting study, and only 9% make their study protocols publicly available. Kolominsky-Rabas et al. [16] noted that three studies had not published any protocols. Study protocols must be published in advance to better assess the potential for bias through selective reporting. Kolominsky-Rabas et al. [16] also noted that registration in a clinical registry alone cannot close the transparency gap. Furthermore, the quality of entries in the clinical registry for DiGA studies must be improved, as they do not meet the Standard Protocol Items: Recommendations for Interventional Trials (SPIRIT) requirements for RCT study protocols [16].

#### 3.2.8. Timeliness (Aspects: Application and Follow-Up Rate and Usage Duration)

The duration of DiGA use in the studies was often shorter than the duration recommended by manufacturers [17]. Albrecht et al. [14] also noted the questionable external validity, with a median duration of use of 3 months and a median follow-up of 6 months in the n = 17 DiGA studies examined. This problem could be addressed by requiring manufacturers to consider “longer-term use under everyday conditions in post-marketing studies” [14].

#### 3.2.9. Consistency (Aspect: Evidence of Medical Benefits/Patient-Relevant Structural or Procedural Improvements)

Four publications noted a trend toward detecting medical benefits, although patient-relevant structural or procedural improvements were used to assess the positive impact on care in only a few studies [16,18,20,25]. Of the 56 DiGAs in the DV at the time, 55 (98%) detected medical benefits, and one (2%) DiGA, which, at that time, was provisionally included, addressed the detection of patient-relevant structural or procedural improvements [18]. Thus, all permanently included DiGAs for detecting the positive impact on care at that time demonstrated medical benefits. Lantzsch et al. [20] described how an increased focus on detecting patient-relevant structural or procedural improvements can lead to better inclusion of real-world data. Further investigation into why patient-relevant structural or procedural improvements are not used more frequently to detect DiGAs’ positive impacts on care could prompt more DiGA manufacturers to adopt them, leading to more patient-centered DiGAs [18]. Groene et al. [26] found that DiGAs can be included on the preliminary list without sufficient evidence of patient-relevant structural or procedural improvements or medical benefits.

## 4. Discussion

### 4.1. Principal Findings

Overall, this scoping review reveals methodological gaps in the quality of evidence regarding DiGA studies. Blinding was lacking in the vast majority of studies. High and heterogeneous dropout rates further undermine reliability. Internal validity is further compromised by the predominant use of passive waiting-list control groups, often combined with self-reported outcomes as primary endpoints, which limit comparability between studies and increase susceptibility to placebo and expectation effects. Concerns regarding relevance and generalizability arise from unclear alignment between intervention goals and medical indications, underrepresentation of certain population groups, and small or variable sample sizes. Finally, shortcomings regarding transparency, including limited availability of full publications and protocols and short follow-up periods, restrict assessment of long-term effectiveness and real-world applicability.

### 4.2. Quality of Evidence

Taken together, the reviewed literature consistently reports methodological limitations in the evidence base supporting permanently listed DiGAs. Future research must address these deficiencies by using transparent reporting, stronger control conditions, more suitable outcome measures that adequately demonstrate the effect, and standardized protocols to ensure more robust and generalizable findings are obtained. The challenges identified for DiGAs are not unique to regulated digital health applications; they reflect broader issues in health app evaluation. Previous research has highlighted similar challenges regarding heterogeneous evaluation criteria, evidence generation, transparency, usability, safety, and implementation aspects across digital health applications in general [36].

The following paragraph critically discusses the findings related to the nine evidence dimensions and derives implications and recommendations.

#### 4.2.1. Objectivity

Overall, the evidence indicates that insufficient blinding is a widespread limitation in DiGA research. Two publications [15,17] emphasized that most trials were effectively open-label, lacking placebo or sham controls, thereby increasing the risk of performance and detection bias. Participants’ awareness of treatment allocation may lead to expectation effects and socially desirable responses, mainly when PROs serve as primary endpoints. Ideally, future trials should incorporate robust blinding strategies, including placebo or sham DiGAs, particularly when using subjective outcome measures. However, limitations regarding blinding and patient-reported outcomes should be interpreted within the specific context of digital health evaluation. Placebo or sham applications are not always feasible or appropriate, and, depending on the research question, pragmatic comparisons with standard care may better reflect real-world effectiveness. Moreover, patient-reported outcomes can serve as meaningful endpoints in digital health for assessing symptoms, quality of life, or patient empowerment. Similarly, dropout rates in digital health studies should be considered not only methodological limitations but also indicators of user engagement and intervention fit, highlighting the need to identify patient groups most likely to benefit from specific digital interventions.

#### 4.2.2. Reliability

Reliability in the DiGA context specifically addresses participant retention and the completeness of outcome data, as high and uneven dropout rates in related studies threaten internal validity and real-world applicability. Overall, the included publications consistently indicate that DiGA studies are affected by high and heterogeneous dropout rates—often higher in the intervention group than in the control—which introduce substantial uncertainty and a risk of bias in the assessment of efficacy. These patterns may reflect usability issues, digital fatigue, or low user engagement, which are common challenges in self-guided digital interventions. To strengthen reliability in future DiGA evaluations, researchers should report dropout rates transparently, implement engagement strategies to reduce attrition, and apply intention-to-treat analyses to minimize bias from missing data. Without these improvements, the robustness and generalizability of DiGA effectiveness data remain limited.

#### 4.2.3. Validity

Validity refers to the extent to which study results reflect actual treatment effects in a manner free from systematic bias caused by poor design, inadequate comparators, or unclear endpoints. In DiGA-related study evaluations, two key threats to internal validity are the widespread use of passive waitlist control groups and the prevalence of subjective PROs as primary endpoints. Most of the studies analyzed (61%) used waitlist controls, while only 33% used standard-therapy comparators, and a small number used active controls. This design choice was criticized in multiple publications [13,14,15,16,17,18,19,20] for potentially inflating treatment effects. Addressing these limitations through active comparators, objective outcome measures, and harmonized endpoint frameworks is essential to strengthen internal validity and support sound regulatory and reimbursement decisions.

#### 4.2.4. Relevance

The clinical relevance of DiGA studies is shaped by how well the intervention aligns with the target indication and how realistically the study design reflects standard care. Waitlist designs, although common, do not mirror standard therapeutic alternatives and may exaggerate treatment effects, especially in mental health studies, which were overrepresented in this group. The literature indicates that DiGA interventions are often complex, leading to challenges in aligning intervention goals with medical indications and to inconsistent assessments of deviations from intended interventions and related risk of bias. On a more positive note, Gregor-Haack et al. [17] report that studies on permanently listed DiGAs tend to feature more thoroughly described interventions, improving clinical relevance. Overall, while the range of indications is appropriate, reliance on passive control groups and variability in reporting of interventions limit the translational value of many DiGA trials.

#### 4.2.5. Generalizability

The generalizability of DiGA study results is limited by insufficient demographic transparency and the underrepresentation of specific population groups, most notably men. Additionally, in many studies, it remains unclear whether the full target population defined by ICD-10-GM criteria was addressed or only if subgroups were included [16]. In sum, limited demographic diversity and poor reporting practices hinder the generalizability of many DiGA trials, underscoring the need for more inclusive study designs and transparent documentation to support equitable digital health access. Beyond DiGAs, several evaluations of digital health applications report similar challenges, including unrepresentative study populations, limited demographic transparency, and restricted external validity, all of which complicate the transferability of results to routine care. In line with our observations, Prentice et al. [36] also identified systematically unrepresentative samples in digital health studies, emphasizing that such sampling issues are not unique to DiGAs but rather reflect a more general problem in the field.

#### 4.2.6. Accuracy

Accuracy concerns the precision of effect estimates, which depends on sample size. The accuracy of clinical trial outcomes is closely linked to sample size, which influences statistical power and the ability to detect actual treatment effects. While there appear to be trends indicating a move toward larger sample sizes [18], a substantial proportion of DiGA studies may lack the sample size needed for robust, generalizable conclusions. While sample sizes appear to increase over time, further studies with larger and more representative samples are needed to improve the precision and robustness of effect estimates.

#### 4.2.7. Transparency

Transparency reflects openness in terms of methods and results as well as accessibility. This element remains a significant concern in the evaluation and implementation of DiGAs. Several studies and stakeholder surveys highlight that healthcare providers and patients lack sufficient information about available DiGAs, their clinical relevance, and practical use. Moreover, registry entries alone are deemed insufficient to address potential biases such as selective reporting. Overall, while peer-reviewed publication rates are relatively high, procedural and reporting transparency remain moderate to low, undermining reproducibility, comparability, and informed clinical use.

#### 4.2.8. Timeliness

Timeliness in clinical studies refers to whether the study duration and follow-up are sufficient to assess meaningful treatment effects. The timeliness of DiGA evaluations—particularly regarding application duration and follow-up—plays a critical role in determining the sustainability of treatment effects. Most DiGAs demonstrate treatment effects in the area of chronic diseases [13]. Chronic diseases are, by definition, long-lasting conditions requiring ongoing care, and evidence suggests that meaningful changes in clinical outcomes may emerge only over extended periods [37]. Consequently, the current literature advocates for using follow-up periods of at least 12 months to reliably evaluate the efficacy and maintenance effects of digital interventions in these populations [37]. If a DiGA is used in a study for less than the necessary duration for adequate treatment, the quality of the evidence may suffer. However, evidence from this scoping review reveals that most studies fail to meet adequate usage and follow-up durations. Gregor-Haack et al. [17] also emphasized that in several cases, the actual usage duration in trials was shorter than that recommended by DiGA manufacturers, potentially leading to an underestimation of real-world efficacy. These limitations highlight the importance of incorporating longer-term observational data, ideally through post-marketing studies, to evaluate DiGAs’ effectiveness in real-world settings over extended periods [14]. Without such data, the evidence base remains skewed toward short-term outcomes, limiting conclusions about clinical durability and long-term benefits. Short follow-up periods should also be considered in the context of the DiGA pathway and digital health market realities. The limited timeframe for generating evidence during provisional listing may create practical barriers to longer follow-up periods, highlighting the need for continued evidence generation after market entry.

#### 4.2.9. Consistency

Overall, while most permanently included DiGAs demonstrate medical benefits, patient-relevant structural or procedural improvements are rarely used to assess positive impacts on care, indicating a gap in patient-centered evaluation despite the potential of this type of evaluation to enhance real-world relevance. For future developments, researchers should therefore aim to strengthen evidence quality without creating disproportionate barriers for digital health innovation. This will require evaluation models that are feasible for all stakeholders and support continuous evidence generation throughout a product’s lifecycle. Real-world evidence, post-market evaluations, and adaptive study approaches may complement traditional trials and help balance timely access to digital health applications with the need for robust evidence on patient benefits [38].

### 4.3. Comparison with International Digital Health Evaluation Frameworks

The methodological limitations identified in this review should be considered within the broader international debate on evidence requirements for digital health technologies. Although Germany’s DiGA framework represents one of the first structured pathways for reimbursement regarding digital therapeutics, similar assessment models have been introduced internationally, including the NHS Digital Technology Assessment Criteria (DTAC) in the United Kingdom, the PECAN pathway in France, mHealthBelgium, and regulatory approaches for software as a medical device by the U.S. Food and Drug Administration (FDA) [39,40,41,42]. While these frameworks differ regarding regulatory scope, assessment criteria, and reimbursement mechanisms (for example, the DTAC focuses not primarily on the feasibility of reimbursement but rather on the holistic trustworthiness of digital health applications, such as technical, medical, and data security [39]), they share the challenge of enabling timely access to digital innovation while ensuring there is sufficient evidence regarding safety and effectiveness. A central finding of this review concerns the selection and validity of endpoints used to demonstrate positive healthcare effects. The frequent use of patient-reported or intermediate outcomes requires careful methodological consideration, particularly in unblinded studies or trials using passive control groups. As highlighted by Fleming and DeMets, surrogate endpoints may not reliably predict meaningful clinical benefits unless their relationship with patient-relevant outcomes has been validated [43]. In line with the GRADE approach, the certainty and applicability of evidence depend not only on study design but also on the relevance of the outcomes, the risk of bias, and the consistency, precision, and directness of the evidence [44]. Future DiGA evaluations should therefore prioritize using predefined endpoints that are clinically meaningful, patient-relevant, and supported by adequate follow-up periods. Where appropriate, patient-reported outcomes should be complemented by objective or behavioral measures to improve the validity and comparability of evidence generated for digital health applications.

### 4.4. Recommendations

To strengthen methodological quality according to the nine dimensions of evidence quality [9], targeted improvements in study design and reporting are required. Addressing the identified gaps would enhance confidence in the demonstrated effects, improve study quality, and allow more robust assessment of patient benefits and real-world applicability.

Various adjustments to the study designs could address these gaps:

Based on the evidence gaps identified, ideally, future DiGA evaluations should prioritize evaluating larger and more representative study populations, using active comparator groups and longer follow-up periods, and improving reporting transparency. Particular emphasis should be placed on implementing appropriate blinding procedures, publishing study protocols before trial initiation, and using outcome measures that enable meaningful assessment of patient benefits under real-world conditions.

By incorporating these considerations into future research, digital health interventions can achieve greater clinical validation, stronger evidence quality, and improved real-world applicability.

However, it is important to note that improving the quality of evidence for digital health applications requires recognizing the specific methodological and practical challenges associated with evaluating digital interventions. Unlike many conventional medical interventions, digital health technologies are characterized by rapid development cycles, frequent updates, changing user interactions, and strong dependency on implementation context [45]. Consequently, evaluation approaches must balance methodological rigor with feasibility, available resources, and the need for timely access to innovation. As highlighted by Njoku et al., digital health evaluations are often constrained by practical barriers, including limited financial resources, recruitment challenges, and difficulties in applying traditional evaluation models to continuously evolving technologies [46].

Therefore, to strengthen the evidence base, researchers should not solely rely on increasing requirements for manufacturers and researchers but also focus on creating supportive structures that enable high-quality evaluations. Adaptive study designs, standardized evaluation frameworks, early methodological guidance, and integration of real-world evidence may help generate robust yet feasible evidence for digital health interventions. The frameworks proposed by Guo et al. and Karpathakis et al. emphasize that successful digital health evaluation requires iterative approaches that combine clinical effectiveness, usability, implementation context, and long-term adoption rather than relying exclusively on traditional efficacy assessment models [47,48]. Future regulatory and reimbursement pathways should therefore be designed to support evidence generation throughout the digital health lifecycle while maintaining appropriate standards for patient benefits and safety.

### 4.5. Limitations

Several limitations should be considered. First, screening, study selection, and data extraction were conducted by a single reviewer, potentially introducing selection bias. To assess the consistency of screening decisions, a retrospective intra-rater reliability assessment was conducted in December 2025. Cohen’s κ = 0.62, indicating substantial agreement. However, because this assessment was conducted retrospectively, it cannot substitute for independent dual-reviewer screening. Therefore, the possibility of selection bias cannot be excluded and should be considered when interpreting the findings of this review. Second, this review focused on permanently listed DiGAs and therefore represents applications that successfully completed the evaluation process. The findings may not fully reflect provisionally listed applications or the broader digital health ecosystem. Additionally, a substantial proportion of the data in this review was derived from early DiGA evaluations [13,14,15,16,17,18,19,20,21,22,23,24,25,26,27,28], which may not fully reflect the current state of the DiGA evidence landscape. An additional limitation is the potential for selection bias arising from restricted search terms and the single-reviewer screening process, which may have led to the exclusion of relevant publications and, consequently, the omission of important insights. Additionally, no statistical analyses were performed in this review, as most of the studies included used a qualitative, narrative, or descriptive approach. The inclusion of different types of publications, including opinion papers and regulatory analyses, introduces heterogeneity in the available evidence. However, this broader inclusion approach was chosen to capture the overall discussion on evidence quality within the DiGA ecosystem. Furthermore, as this review relies on publicly available publications, the possibility of publication and reporting bias cannot be excluded. Furthermore, we did not employ a standardized data extraction template. Given the considerable heterogeneity of the included study types (ranging from narrative reviews and structured assessments to cross-sectional analyses), the use of a uniform template was not feasible. We acknowledge this as a methodological gap and note that this limitation is consistent with the guidance highlighted in the PRISMA-ScR framework. Instead of a fixed template, we organized data extraction according to the nine evidence quality dimensions proposed by Spencer et al., which provided a structured and transparent framework for synthesizing the diverse body of evidence. Finally, a further limitation is the use of the Spencer framework [9], which was originally developed for qualitative evaluation research, not specifically for digital health technologies. Although its broad evidence-quality dimensions provided a useful structure for this review, digital health-specific aspects such as continuous software development, user engagement, implementation processes, and lifecycle-based evidence generation may not be fully represented.

The quality of this scoping review was assessed using the PRISMA-ScR checklist [11]. The review fulfilled the majority (n = 20) of the 22 PRISMA-ScR items. Gaps existed in the provision of a pre-registered study protocol and the presentation of the methodological synthesis of the results (see Appendix B).

## 5. Conclusions

In conclusion, the available literature suggests that the evidence base supporting permanently listed DiGAs is characterized by recurring methodological limitations. Strengthening the evidence base for DiGAs will require approaches that balance methodological rigor with the practical realities of digital health development. Sustainable integration into routine care depends on feasible evaluation pathways that support all stakeholders, including continuous evidence generation, real-world evidence approaches, and structures that address the financial and operational challenges of high-quality digital health evaluations [46].

## Figures and Tables

**Table 1:** Characteristics of the publications included.

No	Study	Publication Year	Publication Type	Thematic Focus	Number of DiGA-Relevant Primary Studies
1	Schreiter S, Mascarell-Maricic L, Rakitzis O et al. [13]	2023	Scoping Review	DiGA Studies	44
2	Albrecht UV, Jan UV, Lawin D et al. [14]	2023	Structured Assessment	DiGA Studies	17
3	König IR, Mittermaier M, Sina C et al. [15]	2022	Narrative Review	Practical Use of DiGA	3
4	Kolominsky-Rabas PL, Tauscher M, Gerlach R et al. [16]	2022	Structured Assessment	DiGA Studies	9
5	Gregor-Haack J, Busse T, Hagenmeyer EG [17]	2021	Narrative Review	DiGA Fast-Track Assessment	1
6	Goeldner M, Gehder S [18]	2024	Cross-Sectional analysis	DiGA Market and Evidence Analysis	0
7	Mäder M, Timpel P, Schönfelder T et al. [19]	2023	Structured Assessment	DiGA Studies	20
8	Lantzsch H, Eckhardt H, Campione A et al. [20]	2022	Structured Assessment	DiGA Studies	16
9	Chalmers M, Hughes J, Rai D, Saini R [21]	2023	Cross-Sectional analysis	DiGA Studies	-
10	Gerlinger G, Mangiapane N, Sander J [22]	2021	Opinion/Position Paper	DiGA Market and Evidence Analysis	0
11	Fujita-Rohwerder N, Sauerland S [23]	2022	Structured Assessment	DiGA Studies	-
12	Gensorowsky D, Witte J, Batram M et al. [24]	2022	Narrative Review	DiGA Market and Evidence Analysis	0
13	Koerber F, Schneider B, Kreuzenbeck C et al. [25]	2023	Structured Assessment	DiGA Studies	-
14	Groene N, Schneck L [26]	2023	Narrative Review	DiGA Reimbursement Policy	1
15	Geier AS [27]	2021	Opinion/Position Paper	DiGA Market and Evidence Analysis	0
16	Dahlhausen F, Zinner M, Bieske L et al. [28]	2021	Empirical Mixed-Methods Study	DiGA Market Analysis	4

**Table 2:** Dimensions of evidence quality.

Dimension and Definition	Aspect	Aspect Reasoning
Objectivity (OBJ)	Blinding	Blinding minimizes assessment bias and strengthens objectivity by preventing expectations from influencing outcomes [29].
Minimization of researcher bias through reflexive practices [9]		
Reliability (RIL)	Dropout rates	A low dropout rate enhances reliability by ensuring that results reflect the outcomes of a stable and consistent participant group [30].
Consistency and dependability of data and methods [10]		
Validity (VAL)	Control group	Including an appropriate control group supports internal validity by enabling meaningful comparisons between intervention and non-intervention outcomes [31].
Credibility of findings in reflecting participants’ perspectives [9]	Primary endpoint	An appropriate primary endpoint ensures a study is measuring what it intends to measure [31].
Relevance (REL)	Indication	Matching the medical indication with the intervention’s goal ensures that a study addresses a meaningful and appropriate clinical question [6].
Fit between the evidence and the research/policy questions [9]		
Generalizability (GEN)	Sample composition	A diverse and representative sample improves generalizability, making findings more applicable to broader patient groups [32].
Transferability of findings to other contexts or settings [9]	Study participants	Demographics and characteristics affect how well results apply to broader populations [32].
Accuracy (ACC)	Sample size	A sufficiently large sample increases the statistical power and accuracy of a study’s effect estimates [33].
Correct and precise representation of data [9]		
Transparency (TRA)	Mandatory publication	This practice reduces publication bias and ensures both positive and negative results are reported, supporting a fuller evidence base [34].
Clarity in reporting methods, decisions, and analysis [9]		
Timeliness (TIM)	Application and follow-up rate	High follow-up and application rates indicate timely and up-to-date data collection, increasing the practical relevance of evidence [35].
Usefulness and relevance of evidence at the time of decision-making [9]	Usage duration	The duration of an intervention may affect findings’ applicability over time.
Consistency (CON)	Evidence of medical benefits/ patient-relevant structural or procedural improvements	Demonstrating consistent improvements across multiple types of outcomes—both medical benefits and patient-relevant structural or procedural improvements—increases the consistency and robustness of evidence [34].
Consistency and dependability of data and methods [9]		

**Table 3:** Assignment of included publications to the nine evidence-quality dimensions. The (✓) symbol indicates that the publication addressed the respective dimension. Assignment to multiple dimensions was possible.

Study	OBJ	RIL	VAL	REL	GEN	ACC	TRA	TIM	CON
Schreiter S, Mascarell-Maricic L, Rakitzis O, Volkmann C, Kaminski J, Daniels MA [13]	✓	✓	✓		✓				
Albrecht UV, Jan UV, Lawin D, Pustozerov E, Dittrich F [14]	✓	✓	✓			✓		✓	
König IR, Mittermaier M, Sina C, Raspe M, Stais P, Gamstaetter T et al. [15]	✓		✓	✓					
Kolominsky-Rabas PL, Tauscher M, Gerlach R, Perleth M, Dietzel N [16]	✓	✓	✓	✓	✓		✓		✓
Gregor-Haack J, Busse T, Hagenmeyer EG [17]	✓	✓	✓	✓				✓	
Goeldner M, Gehder S [18]			✓		✓	✓	✓		✓
Mäder M, Timpel P, Schönfelder T, Militzer-Horstmann C, Scheibe S, Heinrich R et al. [19]	✓	✓	✓		✓				
Lantzsch H, Eckhardt H, Campione A, Busse R, Henschke C [20]			✓	✓			✓		✓
Chalmers M, Hughes J, Rai D, Saini R [21]						✓			
Gerlinger G, Mangiapane N, Sander J [22]							✓		
Fujita-Rohwerder N, Sauerland S [23]			✓						
Gensorowsky D, Witte J, Batram M, Greiner W [24]							✓		
Koerber F, Schneider B, Kreuzenbeck C, Brenner S [25]						✓			✓
Groene N, Schneck L [26]									✓
Geier AS [27]							✓		
Dahlhausen F, Zinner M, Bieske L, Ehlers JP, Boehme P, Fehring L [28]							✓		

Legend: OBJ = objectivity, RIL = reliability, VAL = validity, REL = relevance, GEN = generalizability, ACC = accuracy, TRA = transparency, TIM = timeliness, and CON = consistency.

## Data Availability

No new datasets were generated during this study. The data supporting the findings are derived from publicly available publications, which are cited in the References Section of this manuscript.

## Abbreviations

The following abbreviations are used in this manuscript:

DiGAs	Digitale Gesundheitsanwendungen
DVG	Digitale-Versorgung-Gesetz
SGB	Sozialgesetzbuch
DV	DiGA directory (DiGA-Verzeichnis)
BfArM	Bundesinstitut für Arzneimittel und Medizinprodukte
ICD-10-GM	International Statistical Classification of Diseases and Related Health Problems 10th Revision German Modification
RoB2	Revised Cochrane risk-of-bias tool for randomized trials
RCT	Randomized controlled trial
PRO	Patient-reported outcome
MDR	Medical device regulation

## Appendix A. Inclusion Criteria

**Table A1:** Inclusion criteria.

Topic	Inclusion Criteria
Target Population	Digital health applications listed in the DV
Topic	quality of evidence
Language	German, English
Year	2020–2024
Study type	Review papers
Other factors	Publications providing an overview of the strength of evidence regarding the positive impact on care evaluated in studies related to permanently listed DiGAs

## Appendix B. PRISMA-ScR

**Table A2:** Preferred Reporting Items for Systematic Reviews and Meta-Analyses Extension for Scoping Reviews (PRISMA-ScR) Checklist.

SECTION	ITEM	PRISMA-ScR CHECKLIST ITEM	REPORTED ON PAGE #
**TITLE**			
Title	1	Identify the report as a scoping review.	1
**ABSTRACT**			
Structured summary	2	Provide a structured summary that includes (as applicable): background, objectives, eligibility criteria, sources of evidence, charting methods, results, and conclusions that relate to the review questions and objectives.	1
**INTRODUCTION**			
Rationale	3	Describe the rationale for the review in the context of what is already known. Explain why the review questions/objectives lend themselves to a scoping review approach.	2–3
Objectives	4	Provide an explicit statement of the questions and objectives being addressed with reference to their key elements (e.g., population or participants, concepts, and context) or other relevant key elements used to conceptualize the review questions and/or objectives.	4
**METHODS**			
Protocol and registration	5	Indicate whether a review protocol exists; state if and where it can be accessed (e.g., a Web address); and if available, provide registration information, including the registration number.	5
Eligibility criteria	6	Specify characteristics of the sources of evidence used as eligibility criteria (e.g., years considered, language, and publication status), and provide a rationale.	6–7
Information sources *	7	Describe all information sources in the search (e.g., databases with dates of coverage and contact with authors to identify additional sources), as well as the date the most recent search was executed.	5
Search	8	Present the full electronic search strategy for at least 1 database, including any limits used, such that it could be repeated.	5
Selection of sources of evidence ^†^	9	State the process for selecting sources of evidence (i.e., screening and eligibility) included in the scoping review.	5–6
Data charting process ^‡^	10	Describe the methods of charting data from the included sources of evidence (e.g., calibrated forms or forms that have been tested by the team before their use, and whether data charting was done independently or in duplicate) and any processes for obtaining and confirming data from investigators.	5–6
Data items	11	List and define all variables for which data were sought and any assumptions and simplifications made.	8–10
Critical appraisal of individual sources of evidence ^§^	12	If done, provide a rationale for conducting a critical appraisal of included sources of evidence; describe the methods used and how this information was used in any data synthesis (if appropriate).	n.a.
Synthesis of results	13	Describe the methods of handling and summarizing the data that were charted.	5–6
**RESULTS**			
Selection of sources of evidence	14	Give numbers of sources of evidence screened, assessed for eligibility, and included in the review, with reasons for exclusions at each stage, ideally using a flow diagram.	7
Characteristics of sources of evidence	15	For each source of evidence, present characteristics for which data were charted and provide the citations.	7–8
Critical appraisal within sources of evidence	16	If done, present data on critical appraisal of included sources of evidence (see item 12).	n.a.
Results of individual sources of evidence	17	For each included source of evidence, present the relevant data that were charted that relate to the review questions and objectives.	10–16
Synthesis of results	18	Summarize and/or present the charting results as they relate to the review questions and objectives.	7–16
**DISCUSSION**			
Summary of evidence	19	Summarize the main results (including an overview of concepts, themes, and types of evidence available), link to the review questions and objectives, and consider the relevance to key groups.	16–21
Limitations	20	Discuss the limitations of the scoping review process.	22
Conclusions	21	Provide a general interpretation of the results with respect to the review questions and objectives, as well as potential implications and/or next steps.	22–23
**FUNDING**			
Funding	22	Describe sources of funding for the included sources of evidence, as well as sources of funding for the scoping review. Describe the role of the funders of the scoping review.	23

JBI = Joanna Briggs Institute; PRISMA-ScR = Preferred Reporting Items for Systematic reviews and Meta-Analyses extension for Scoping Reviews. * Where *sources of evidence* (see second footnote) are compiled from, such as bibliographic databases, social media platforms, and Websites. ^†^ A more inclusive/heterogeneous term used to account for the different types of evidence or data sources (e.g., quantitative and/or qualitative research, expert opinion, and policy documents) that may be eligible in a scoping review as opposed to only studies. This is not to be confused with *information sources* (see first footnote). ^‡^ The frameworks by Arksey and O’Malley (6) and Levac and colleagues (7) and the JBI guidance (4, 5) refer to the process of data extraction in a scoping review as data charting. ^§^ The process of systematically examining research evidence to assess its validity, results, and relevance before using it to inform a decision. This term is used for items 12 and 19 instead of “risk of bias” (which is more applicable to systematic reviews of interventions) to include and acknowledge the various sources of evidence that may be used in a scoping review (e.g., quantitative and/or qualitative research, expert opinion, and policy document). *From:* Tricco AC, Lillie E, Zarin W, O’Brien KK, Colquhoun H, Levac D, et al. PRISMA Extension for Scoping Reviews (PRISMAScR): Checklist and Explanation. Ann Intern Med. 2018;169:467–473. doi: 10.7326/M18-0850.
